# Lung ultrasound score and diaphragm ultrasound in weaning from mechanical ventilation: are they different in patients with and without COVID-19?

**DOI:** 10.36416/1806-3756/e20240302

**Published:** 2024-11-16

**Authors:** Laura Cordeiro Madeira, Paulo de Tarso Dalcin, Gabriele Heinen Schuster, Bruna Conte, Jonas Michel Wolf, Annia Schreiber, Jean-Jacques Rouby, Felippe Leopoldo Dexheimer-Neto

**Affiliations:** 1. Centro de Terapia Intensiva, Hospital Moinhos de Vento, Porto Alegre (RS) Brasil.; 2. Programa de Pós-Graduação em Ciências Pneumológicas, Hospital de Clínicas de Porto Alegre, Universidade Federal do Rio Grande do Sul, Porto Alegre (RS) Brasil.; 3. Serviço de Pneumologia, Hospital de Clínicas de Porto Alegre, Universidade Federal do Rio Grande do Sul, Porto Alegre (RS) Brasil.; 4. Escritório de Gestão da Prática Clínica, Hospital Moinhos de Vento, Porto Alegre (RS) Brasil.; 5. Unity Health Toronto, St. Michael’s Hospital, Interdepartmental Division of Critical Care Medicine, Toronto (ON) Canada.; 6. Réanimation Polyvalente, Département d’Anesthésie-Réanimation, Universitè Pierre et Marie Curie - UPMC - Sorbonne Université, Paris, France.

**Keywords:** COVID-19, Ventilator weaning, Diaphragm, Ultrasound/diagnosis, Lung

## Abstract

**Objective:**

To compare pre-extubation physiological characteristics and ultrasound variables between patients intubated for COVID-19 compared to a clinical population and those intubated for other reasons.

**Methods:**

This was a secondary analysis of a prospective cohort study of patients undergoing invasive mechanical ventilation (IMV) for more than 48 h. Patients were divided into two groups: those intubated for COVID-19-induced ARDS and those intubated for other clinical reasons. Ultrasound assessment of lung and diaphragm function was performed before extubation. The results were compared between the two groups of patients.

**Results:**

In comparison with the patients without COVID-19, those with the disease were younger (a median age of 58 [46-76] years vs. a median age of 75 [69-85] years; p = 0.01), had fewer comorbidities (a median Charlson Comorbidity Index of 2 [1-4] vs. a median Charlson Comorbidity Index of 5 [4-6]; p < 0.01), and were less severely ill at admission (a median APACHE II score of 9 [8-14] vs. a median APACHE II score of 18 [13-22]; p < 0.01). In addition, the median duration of IMV was longer in the COVID-19 patients (11 [9-23] days vs. 6 [3-8] days; p < 0.01). Although extubation success rates were similar between the COVID-19 and non-COVID-19 groups (22 [71%] vs. 35 [77.8%]), median lung ultrasound score differed between the two groups (23 [18-25] vs. 15 [11-18]; p < 0.01), as did median diaphragmatic excursion (2.1 [1.7-2.4] vs. 1.7 [1.2-2.0]; p < 0.01).

**Conclusions:**

Although patients with COVID-19 requiring ventilatory support are younger and have fewer comorbidities than those intubated for other clinical reasons, they experience longer hospital stays. Although lung ultrasound score can differ between patients with and without COVID-19, these differences do not significantly translate into extubation success rates. Therefore, the utility of ultrasound scores in weaning COVID-19 patients from IMV needs further study.

## INTRODUCTION

The COVID-19 pandemic, caused by SARS-CoV-2, has posed an unprecedented challenge to health care systems worldwide.^1^ ^,^ ^2^ In addition to the impact of COVID-19 on public health, the treatment of patients with severe COVID-19, which often leads to ARDS, has been a major focal point. Invasive mechanical ventilation (IMV) has become a crucial intervention for many COVID-19 patients who develop severe acute respiratory failure, such patients accounting for approximately 20% of all hospitalized COVID-19 patients.^3^ ^,^ ^4^ However, successful weaning from IMV and extubation pose significant challenges, given the complexity of the disease and its specific complications. Extubation failure rates in this population appear to be as high as 40%,^5^ resulting in high morbidity and mortality.^6^ ^,^ ^7^

Weaning from mechanical ventilation in COVID-19 patients can be particularly challenging because COVID-19 causes severe lung inflammation, blood clot formation, pulmonary fibrosis, and muscle weakness. These complications can prolong the need for ventilatory support. Therefore, weaning strategies must be adapted to meet the needs of COVID-19 patients, ensuring a safe transition to spontaneous breathing and minimizing the risk of relapse or reintubation. Lung ultrasound has proven to be a valuable tool in the diagnosis and prognosis of patients with COVID-19, helping to identify those at a higher risk of progression to IMV.^8^ ^-^ ^10^ However, the use of lung ultrasound in weaning from ventilatory support has yet to be clarified. 

In this context, the objective of the present study was to compare pre-extubation physiological characteristics and ultrasound variables between patients intubated for COVID-19 and those intubated for other reasons in the same period. 

## METHODS

This was a secondary analysis of an original study evaluating the accuracy of thoracic ultrasound in predicting extubation success in patients on IMV for more than 48 h. Because part of the study sample consisted of patients with COVID-19 pneumonia, we decided to compare the patients with and without COVID-19 in terms of physiological characteristics and ultrasound findings. 

A prospective cohort study was conducted between January of 2021 and April of 2023 in the ICU of *Hospital Moinhos de Vento*, located in the city of Porto Alegre, southern Brazil. The ICU comprises 72 beds for clinical and surgical admissions. The study was approved by the local research ethics committee (Protocol no. 21991519.8.0000.5330) and was conducted in accordance with the Declaration of Helsinki. All responsible parties of the participating patients gave written informed consent.

The inclusion criteria were as follows: being > 18 years of age; and having been on IMV for more than 48 h because of COVID-19 pneumonia or other causes, the condition leading to IMV being resolved or controlled. After a successful spontaneous breathing trial (SBT) and prior to extubation, thoracic ultrasound was performed, including an assessment of lung aeration and diaphragm function. The exclusion criteria were as follows: having a tracheostomy; receiving exclusive palliative care; having advanced pulmonary fibrosis; having end-stage neuromuscular disease; being on home IMV; being pregnant or lactating; having previously failed extubation during the same hospitalization; and being clinically unable to undergo ultrasound examination. The SBT was performed either with a T-tube or on pressure support mode with reduced parameters. 

Arterial blood gas data and vital signs were collected on the day of extubation. The following were also assessed: duration of ventilation until successful SBT; rate of tracheostomy; use of vasoactive drugs; need for renal replacement therapy; and mortality. 

### Thoracic ultrasound

Ultrasound evaluation was performed by two intensivists with experience in thoracic ultrasound. Calibration was achieved through 20 simultaneous examinations and was assessed with Pearson’s correlation coefficient. Thoracic ultrasound, including assessment of lung aeration and diaphragm function, was performed at the end of the SBT, prior to extubation, with the patient in the supine position, with the head of the bed elevated at 30-45°. 

Lung ultrasound was performed with a 2-4 MHz convex probe. To calculate the lung aeration score, the anterior, lateral, and posterior areas of the upper and lower intercostal spaces were examined, totaling 12 regions. Four lung aeration patterns were evaluated: normal aeration (0), characterized by pleural sliding with A-lines or a few B-lines (a maximum of 2); moderate loss of lung aeration (1), characterized by multiple well-defined B-lines; significant loss of lung aeration (2), characterized by multiple coalescent B-lines; and lung consolidation (3), characterized by complete loss of aeration. The lung ultrasound score (LUS) was calculated on the basis of the worst observed pattern and ranged from 0 to 36.^11^

Ultrasound assessment of diaphragm function included assessment of diaphragmatic excursion (DE) and diaphragm thickening fraction (DTF) on the right hemidiaphragm, the best ultrasound window being considered. To assess DE, a 2-4 MHz convex probe was used. The probe was placed in the lowest intercostal spaces, on the right anterior axillary line, with the liver as a window for imaging. Initially, the two-dimensional mode was used in order to determine the best approach and select the scanning line of the hemidiaphragm. The ultrasound beam was directed to the diaphragmatic dome at an angle of approximately 70°, measurements being then performed in M-mode. The excursion amplitude was measured on the vertical axis of the trace, from the baseline to the point of maximum height during inspiration. The average of three measurements was calculated.^12^ DTF was assessed with a 5-7 MHz linear probe and measured at the zone of apposition of the diaphragm and the rib cage between the anterior axillary and midaxillary lines, between the eighth and tenth intercostal spaces. The two-dimensional mode was used in order to locate the best image, measurements being then performed in M-mode. Diaphragm thickness was evaluated at the end of inspiration (DTi) and at the end of expiration (DTe), DTF being calculated by the following formula and expressed as a percentage: (DTi − DTe)/DTe × 100.^13^ The average of three measurements was calculated. 

### Statistical analysis

Data were collected and analyzed with the IBM SPSS Statistics software package, version 25.0 (IBM Corporation, Armonk, NY, USA), and R software, version 4.3.0 (The R Foundation for Statistical Computing, Vienna, Austria). Qualitative variables were expressed as absolute and relative frequencies, whereas quantitative variables were expressed as medians and interquartile ranges. For quantitative variables, the data distribution types were assessed with the Shapiro-Wilk test, and the Mann-Whitney U test was used. Multivariate Poisson regression was performed to adjust for potential influences of covariates. 

## RESULTS

**Figure 1:**
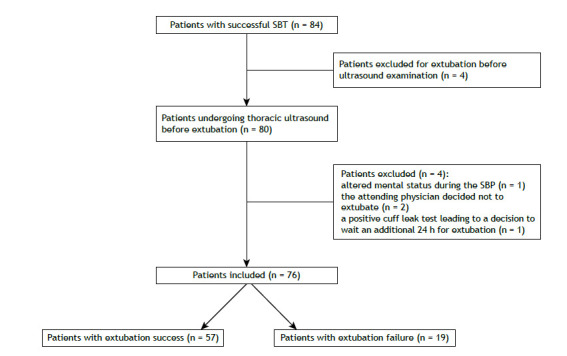
Flow chart of patient selection. SBT: spontaneous breathing trial.

Seventy-six patients on IMV for more than 48 h were included in the study. Figure 1 shows the patient selection process. The patients with COVID-19 were younger than those without the disease (a median age of 58 [46-76] years vs. a median age of 75 [69-85] years; p = 0.01), had fewer comorbidities (a median Charlson Comorbidity Index of 2 [1-4] vs. a median Charlson Comorbidity Index of 5 [4-6]; p < 0.01), and were less severely ill at ICU admission (a median APACHE II score of 9 [8-14] vs. a median APACHE II score of 18 [13-22]; p < 0.01). The patients intubated for COVID-19 spent more time on IMV until SBT (10 [7-13] days vs. 5 [3-8] days; p < 0.01) and underwent SBT more often on pressure support ventilation (29 [93.5%] vs. 11 [24.4%]; p = 0.01) than did those intubated for other reasons. There was no difference in extubation success within 72 h between the non-COVID-19 and COVID-19 groups (35 [77.8%] vs. 22 [71%]; p = 0.5). However, the non-COVID-19 population had more patients with simple weaning (34 [75.6%] vs. 13 [41.9%]; p = 0.003) and underwent fewer tracheostomies (5 [11.1%] vs. 9 [29%]; p = 0.04). On the day of the SBT and subsequent extubation, median PaCO_2_ was slightly higher in the patients with COVID-19 than in those without the disease (42 [40-48] vs. 39 [36-44]; p = 0.02). There was no difference in mortality between the non-COVID-19 and COVID-19 patients (16 [35.6%] vs. 6 [20%]; p = 0.14). Table 1 shows the characteristics of the two populations. 

**Table 1:** Demographic characteristics of the study sample.^a^

Variable	Total	Non-COVID-19	COVID-19	p	
				Crude*	Adjusted**
Number of patients	76 (100%)	45 (59.2%)	31 (40.8%)		
Age, years	71 (56-82)	75 (69-85)	58 (46-76)	0.01	0.03
Male	46 (60.5%)	26 (57.8%)	20 (64.5%)	0.55	0.49
Charlson Comorbidity Index	4 (2-6)	5 (4-6)	2 (1-4)	< 0.01	0.04
APACHE II score	14 (9-20)	18 (13-22)	9 (8-14)	< 0.01	< 0.01
Pre-ICU admission length of stay, days	1 (0-4)	1 (0-5)	2 (1-4)	0.54	0.63
Duration of IMV before SBT, days	7 (4-10)	5 (3-8)	10 (7-13)	< 0.01	0.01
PaCO_2_, mmHg	41 (37-46)	39 (36-44)	42 (40-48)	0.02	0.08
Duration of SBT, min	50 (30-60)	50 (35-60)	60 (30-69)	0.18	0.23
Use of vasopressor	74 (97.4%)	44 (97.8%)	30 (96.8%)	> 0.99	0.94
Need for RRT	21 (27.6%)	15 (33.3%)	6 (19.4%)	0.18	0.12
Systemic corticosteroid use	63 (82.9%)	32 (71.1%)	31 (100%)	0.01	0.01
SBT on PSV	40 (52.5%)	11 (24.4%)	29 (93.5%)	< 0.01	< 0.01
Extubation success within 72 h	57 (75%)	35 (77.8%)	22 (71%)	0.50	0.47
Tracheostomy	14 (18.4%)	5 (11.1%)	9 (29%)	0.04	0.04
Simple weaning	47 (61.8%)	34 (75.6%)	13 (41.9%)	< 0.01	< 0.01
Duration of IMV, days	8 (4-12)	6 (3-8)	11 (9-23)	< 0.01	< 0.01
Length of ICU stay, days	17 (12-29)	15 (10-23)	23 (16-36)	< 0.01	< 0.01
Length of hospital stay, days	35 (21-51)	32 (18-51)	38 (23-57)	< 0.01	0.03
Death during hospitalization	22 (29.3%)	6 (20%)	16 (35.6%)	0.14	0.08
Discharge from the ICU	57 (75%)	25 (80.6%)	32 (71.1%)	0.34	0.31

IMV: invasive mechanical ventilation; SBT: spontaneous breathing trial; RRT: renal replacement therapy; and PSV: pressure support ventilation. ^a^Data expressed as n (%) or median (IQR). *Pearson’s chi-square test or Fisher’s exact test for qualitative variables and the Mann-Whitney test for quantitative variables. **Multivariate Poisson regression to adjust for potential influences of covariates.

**Figure 2:**
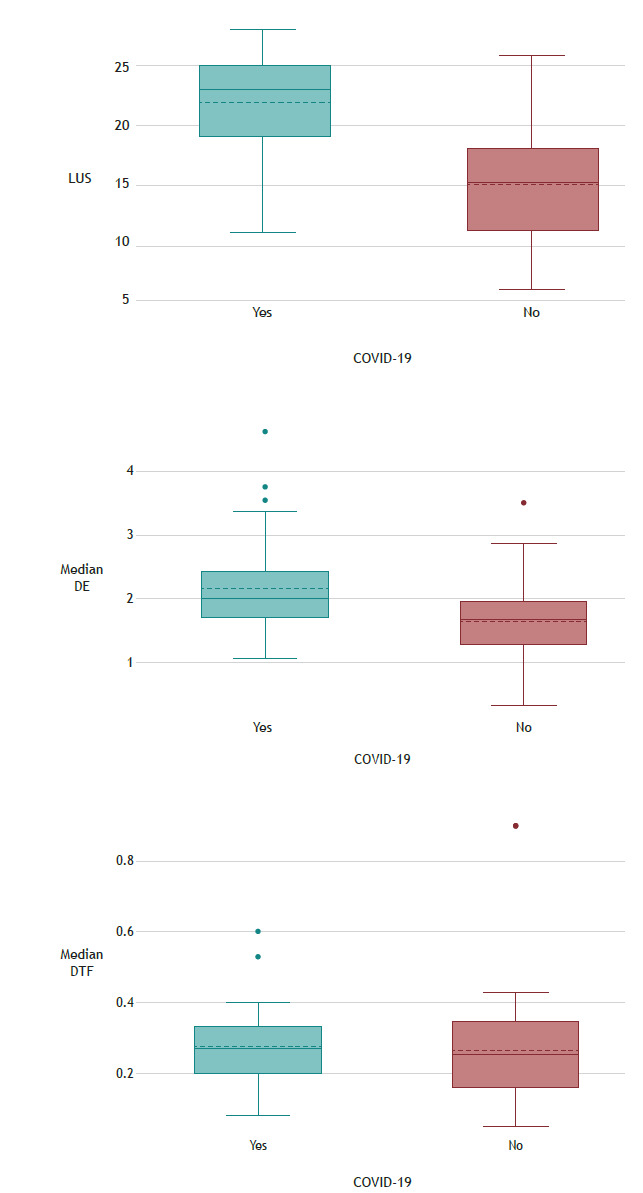
Box plot of ultrasound variables in patients with and without COVID-19. LUS: lung ultrasound score; DE: diaphragmatic excursion; and DTF: diaphragm thickening fraction.

Inter-rater reliability was found to be good, with an I^2^ of 0.83 for the LUS, an I^2^ of 0.82 for DE, and an I^2^ of 0.87 for DTF. With regard to the results of ultrasound assessment (Table 2 and Figure 2), even after a multivariate analysis, *correcting for variables that showed significant differences,* the median LUS of the COVID-19 patients was significantly higher than that of the non-COVID-19 patients (23 [18-25] vs. 15 [11-18]; p < 0.01), as was the median DE (2.1 [1.7-2.4] vs. 1.7 [1.3-2.0]; p < 0.01). As can be seen in Table 2 and Figure 2, DTF was similar between the two groups of patients (30% [20-40%] vs. 30% [20-30%]; p = 0.49). 

**Table 2:** Multivariate analysis of ultrasound variables in patients with and without COVID-19.^a^

Variable	Total	COVID-19	Non-COVID-19	p*	
				Crude	Adjusted
LUS	17 (13-24)	23 (18-25)	15 (11-18)	< 0.01	0.02
DE, cm	1.76 (1.42-2.22)	2.1 (1.7-2.4)	1.7 (1.3-2.0)	< 0.01	0.04
DTF, %	26 (16-35)	30 (20-30)	30 (20-40)	0.49	0.45

LUS: lung ultrasound score; DE: diaphragmatic excursion; and DTF: diaphragm thickening fraction. ^a^Data expressed as median (IQR). *Mann-Whitney test, crude and adjusted for variables showing significant differences in the multivariate Poisson regression model.

A binary logistic regression evaluating the subgroup of patients intubated for COVID-19 showed that the ultrasound scores were not accurate in predicting extubation success (Table 3). 

**Table 3:** Ultrasound evaluation of COVID-19 patients who failed or succeeded extubation within 72 h.^a^

	Total	Extubation failure	Extubation success	Crude		Adjusted*	
				OR (95% CI)	p	OR (95% CI)	p
LUS	23(19-25)	25 (24-26)	21 (18-24)	0.88 (0.68-1.08)	0.26	0.82 (0.6-1.02)	0,10
DE, cm	2.01 (1.73-2.41)	2.13 (1.75-2.39)	1.86 (1.68-2.38)	0.73 (0.31-2.53)	0.74	0.7 (0.19-2.7)	0,57
DTF, %	27 (20-33)	28 (18-34)	26 (21-32)	1 (0.93-1.08)	0.96	1.02 (0.94-1.12)	0,65

LUS: lung ultrasound score; DE: diaphragmatic excursion; and DTF: diaphragm thickening fraction. ^a^Data expressed as median (IQR). *Binary logistic regression adjusted for potential influences of covariates.

## DISCUSSION

In the present study we compared patients intubated for COVID-19 with those intubated for other reasons. The group of patients with COVID-19 was younger and had fewer comorbidities. They also had a lower severity score at ICU admission. However, they remained on IMV for a longer duration of time, resulting in longer ICU and hospital stays. Nevertheless, they did not have higher mortality rates. Socolovithc et al. reported similar findings in a study in which patients with COVID-19 required IMV three times more often than did those admitted for other reasons.^14^ A systematic review including 32 studies and over 69,000 patients confirmed these findings, showing high rates of IMV use, longer lengths of stay, and elevated mortality.^15^

Lung ultrasound has been used in order to diagnose and prognosticate COVID-19 pneumonia.^8^ ^,^ ^9^ However, only a few studies have examined the use of lung ultrasound in weaning from IMV. This is the first study to assess the LUS in COVID-19 patients at the time of extubation. The LUS is used as a tool to aid in weaning non-COVID-19 patients, with cutoff points of 13 or less to predict successful weaning from IMV.^11^ ^,^ ^16^ In the present study, the patients with COVID-19 had a higher LUS than did those without the disease; however, they achieved similar extubation success rates, indicating that a cutoff point of 13 or less may not be applicable to these individuals. The role of LUS in weaning from IMV still needs further investigation. In assessing the ability of ultrasound scores to predict extubation success in patients with COVID-19, the LUS tended to be higher in those in whom extubation failed, although the difference was not significant. This could be explained by the small number of intubated patients with COVID-19. 

The diaphragm also appears to be affected differently in this population of patients. In a study involving autopsy diaphragm specimens from 34 critically ill individuals (26 of whom had COVID-19 and 8 of whom did not have the disease), Shi et al. demonstrated that fibrosis was twice as high in those with COVID-19 than in those without the disease, suggesting that severe COVID-19-induced myopathy leads to diaphragmatic weakness and contributes to weaning failure.^17^ seems to be a predictor of worse prognosis, associated with lymphocyte count.^18^ Hadda et al. found that DTe decreases during hospitalization, although the study population consisted of spontaneously breathing patients.^19^ Corradi et al. showed that DTF may be a predictor of failure of noninvasive support in COVID-19 patients.^20^ However, the use of DTF in weaning from IMV has been poorly studied. Vetrugno et al. evaluated DTF as an indicator of weaning failure in patients with COVID-19 but found no difference between the success and failure groups.^5^ In the present study, DTF was similar between the groups of patients with and without COVID-19. In addition, our subgroup analysis of patients intubated for COVID-19 showed that DTF was not a good predictor of extubation success. 

Regarding the assessment of DE in COVID-19 patients, it has been shown that DE assessment within the first 15 min of SBT has good accuracy in predicting weaning success even when performed under positive pressure, where it may be influenced by increased lung volumes.^21^ ^,^ ^22^ In the present study, DE was higher in the COVID-19 group than in the non-COVID-19 group; however, DE was not a good predictor of extubation success when evaluated exclusively in the group of patients with COVID-19. In one of the aforementioned studies,^21^ pressure support ventilation during SBT was set at 5 cmH2O, whereas, in the present study, it varied. 

The present study is the first to compare ultrasound scores between patients intubated for COVID-19 and those intubated for other reasons. Additionally, this is the first study to assess the LUS at the time of extubation in patients with SARS-CoV-2 pneumonia. However, the study was conducted at a single center and is a secondary analysis of an original study. 
